# Genome-Wide Identification and Characterization of the ALOG Domain Genes in Rice

**DOI:** 10.1155/2019/2146391

**Published:** 2019-02-24

**Authors:** Na Li, Yang Wang, Jing Lu, Chuan Liu

**Affiliations:** ^1^State Key Laboratory of Hybrid Rice, College of Life Sciences, Wuhan University, Wuhan 430072, China; ^2^College of Food Science and Technology, Huazhong Agricultural University, 430070 Wuhan, China; ^3^Chongqing Key Laboratory on Big Data for Bio Intelligence, Chongqing University of Posts and Telecommunications, Chongqing 400065, China

## Abstract

The ALOG domain genes, named after the *Arabidopsis* LSH1 and *Oryza* G1 (ALOG) proteins, have frequently been reported as key developmental regulators in rice and *Arabidopsis*. However, the investigation of the ALOG gene family is limited. Here, we conducted a genome-wide investigation of the ALOG gene family in rice and six other species. In total, eighty-four ALOG domain genes were identified from the seven species, of which fourteen ALOG domain genes (*OsG1*/*G1Ls*) were identified in the rice genome. The fourteen *OsG1*/*G1Ls* were unevenly distributed on eight chromosomes, and we found that eight segmental duplications contributed to the expansion of *OsG1*/*G1Ls* in the rice genome. The eighty-four ALOG family genes from seven species were classified into six clusters, and the ALOG domain-defined motifs 1, 2, and 3 were highly conserved across species according to the phylogenetic and structural analysis. However, the newly identified motifs 4 and 5 were only present in monocots, indicating a specified function in monocots. Moreover, *OsG1*/*G1Ls* exhibited tissue-specific expression patterns. Coexpression analysis suggested that *OsG1* integrates *OsMADS50* and the downstream MADS-box genes, such as *OsMADS1*, to regulate the development of rice inflorescence; *OsG1L7* potentially associates with *OsMADS22* and *OsMADS55* to regulate stem elongation. In addition, comparative expression analysis revealed the conserved biological functions of ALOG family genes among rice, maize, and *Arabidopsis*. These results have shed light on the functional study of ALOG family genes in rice and other plants.

## 1. Introduction

Rice (*Oryza sativa*) is one of the most important crops as a sustenance source to over 3.5 billion individuals of the global population [1]. Unraveling the molecular regulatory mechanisms of diverse agronomic traits is beneficial for rice breeding practice and may be critical for global food security. In addition, rice is an ideal model organism for plant functional genomics research and studies on the evolutionary lineage of monocotyledons. Based on these prerequisites, functional genomics technologies have rapidly been developed and applied for the identification of thousands of genes controlling important agronomic traits in rice. As the online genomic data and the number of cloned genes have increased, the genome-wide analysis of gene families has become a tool to explore the potential roles of the previously uncharacterized genes in rice as well as other commercial crops [2–6].

Rice yield and yield-related traits have been considered important agronomic traits during hybrid breeding practice, particularly grain size, grain number, flowering time, and plant height. Quantitative trait loci (QTL) or genes, such as *Ghd7*, *OsPRR37*, and *DTH8*, were shown to affect rice yield by pleiotropically regulating rice flowering time, plant height, and grain number [7–11]. Other QTL or genes controlling grain size, including *GS3*, *GS5*, *GW2*, *GW5*, *GW7*, and *GL3.3*, can affect rice yield mainly because grain size is typically associated with grain weight [12–17]. Furthermore, a recent study established a G-protein pathway comprising five subunits of the heterotrimeric G proteins that determines the grain size in rice, demonstrating a conserved system among angiosperms to manipulate grain/organ size [18]. Additionally, the increase in the size of lemma/palea could also potentially increase rice yields by increasing the grain size [19]. Therefore, characterization of the genes and gene homologs that regulate the shape of lemma/palea is helpful for genetically manipulating the grain size and yield-related traits.

A single rice spikelet contains a pair of subtended lemma and palea and two sterile lemmas. Several studies have reported the genes involved in the developmental regulation of lemma/palea and sterile lemma in rice. Among these genes, *OsG1* represses the homeotic transformation of the sterile lemma to the regular lemma [20], and *TH1*/*BH1*/*AFD1* affects the development of the lemma/palea, spikelet morphogenesis, grain shape, and yield in rice [21–25]. Coincidentally, these two genes are both DUF640 domain-containing proteins. The DUF640 domain is also known as the ALOG domain, named after the *Arabidopsis* LSH1 and *Oryza* G1 proteins [20, 26, 27]. However, *Arabidopsis LSH1* mediates the light-dependent regulation of the hypocotyl length, appearing to have a function different from that of *OsG1* [26]. In addition, *TAWAWA1*, another homolog of *OsG1*, is a unique regulator of meristem activity in rice and regulates inflorescence development through the promotion of inflorescence meristem activity and the suppression of the phase change to spikelet meristem identity [28]. These results indicated that ALOG domain genes play multiple roles in plant development, especially in the developmental regulation of floral organ identity and inflorescence meristem activity. Genome-wide expression and evolutionary analyses of the ALOG domain gene family in rice and other plant species would help to understand the regulatory mechanism of this gene family; however, these studies are still limited.

In the present study, the ALOG domain genes were first identified from 7 species, including four monocotyledons (*Oryza sativa*, *Zea mays*, *Sorghum bicolor*, and *Brachypodium distachyon*), two dicotyledons (*Arabidopsis thaliana* and *Populus trichocarpa*), and a moss (*Physcomitrella patens*). We primarily investigated the evolutionary relationships, gene duplication, gene structure, and expression profiles of the ALOG domain gene family, particularly the ALOG domain genes (*OsG1*/*G1Ls*) in rice. Based on the results, we attempted to reveal the tissue-specific expression patterns and potential functions of the ALOG family genes in rice as well as in other species. Particularly, the functional relationships between *OsG1*/*G1Ls* and MADS-box genes were deduced by coexpression analyses in rice. In addition, a comparative expression analysis was applied to study the conserved functions of ALOG family genes among rice, maize, and *Arabidopsis*. The present study not only provides theoretical evidence for the functional study of ALOG domain genes but also evokes a further application of the ALOG domain genes in the breeding of high-yield rice and other crops.

## 2. Materials and Methods

### 2.1. Identification of ALOG Domain Genes

Local databases, including protein sequences, coding sequences (CDS), and mRNA sequences, were established by downloading data from the Rice Genome Annotation Project (RGAP, release 7.0, http://rice.plantbiology.msu.edu), the *Arabidopsis* Information Resource (TAIR, release 10, http://www.arabidopsis.org), the Maize Genetics and Genomics Database (MaizeGDB, B73RefGen_v2, http://www.maizegdb.org), and JGI (http://genome.jgi-psf.org). HMMER (http://hmmer.org/) and name searches were performed on the local proteome sequence database by using the Pfam profile PF04852, and the results were then combined with BLAST search results to identify rice and maize ALOG domain-containing genes. Since the ALOG domain was conserved and specific, an *E* value of 1*e* − 10 was used as the cut-off in the HMMER and BLAST searches. Within the search results, we manually removed the number of hits that only had partial ALOG domains. Information on the gene structures, full-length cDNAs, and RAP accession numbers for each gene and the characteristics of the corresponding proteins were procured from RAP-DB, RGAP, and KOME (https://dbarchive.biosciencedbc.jp/en/kome/desc.html). Additionally, the Pfam (http://www.sanger.ac.uk/Software/Pfam/) and InterPro (http://www.ebi.ac.uk/interpro/) protein family databases were used to confirm and classify each predicted ALOG protein.

### 2.2. Chromosomal Location and Gene Duplication Analysis

All *OsG1*/*G1Ls* were mapped onto the chromosomes by identifying their chromosomal positions in the TIGR database, and the segmentally duplicated genes were identified from the Plant Genome Duplication Database (PGDD) with a parameter of 500 kb [29]. The DAGchainer program was then used to confirm the segmental duplications with the parameters *V* = 5, *B* = 5, *E* = 1*e* − 10 filter seg, and distance = 500 kb; genes separated by five or fewer genes were considered tandem duplicates [30]. The Ka and Ks values of the duplicated gene pairs were obtained from the identification results of segmentally duplicated genes in PGDD [29]. The approximate date of occurrence of the duplication events was calculated by the equation *T* = Ks/2*λ*. The mean synonymous substitution rate (*λ*) for rice is 6.5*e* − 9 [31].

### 2.3. Phylogenic and Structural Analysis

For the phylogenetic analysis, we only considered the amino acid sequences of the ALOG domain because no other regions could be unambiguously aligned for all of the available sequences. MUSCLE software was used to align the sequences with the default parameters, and poorly aligned positions and divergent regions were eliminated from the aligned sequences by using Gblocks [32, 33]. A phylogenetic tree was constructed with the default parameters and the aLRT statistical test model using PhyML [34]. Finally, the tree was rendered and displayed with TreeDyn [35], and the branches were annotated with 1000 resamplings for the bootstrap test values. The exon-intron organizations for all genes were obtained by using the online Gene Structure Display Server (GSDS, http://gsds.cbi.pku.edu.cn) with both the predicted coding sequences and the corresponding genomic sequences [36]. The ALOG protein sequences were statistically analyzed with the MEME program (http://meme-suite.org/) to confirm the presence of the conserved motifs using the following parameters: number of repetitions set as “any,” maximum number of motifs of 5, and optimum motif width set to >5 and <150 [37].

### 2.4. Microarray-Based Expression Analysis

All expression profile data, including those for the ALOG domain genes of maize and *OsG1*/*G1Ls*, which cover 60 tissues of B73 and 33 tissues of two *indica* rice varieties Zhenshan 97 (ZS97) and Minghui 63 (MH63), were collected from the PLEXdb (http://www.plexdb.org/) and CREP (http://crep.ncpgr.cn) databases [38, 39]. Then, the massively parallel signature sequencing (MPSS) data were used to determine the expression profiles of the genes with conflicting probe set signals [40]. All *Arabidopsis* microarray data were downloaded from the Gene Expression Omnibus database (http://www.ncbi.nlm.nih.gov/geo) using the GSE series accession numbers GSE5629, GSE5630, GSE5631, GSE5632, GSE5633, and GSE5634. The subsequent analysis of the gene expression data was performed in the statistical computing language R (http://www.r-project.org) using packages available from the Bioconductor project (http://www.bioconductor.org), and the raw data were processed with the Affymetrix Microarray Analysis Suite (MAS version 5, Affymetrix) [41]. The Euclidean distances of all *OsG1*/*G1Ls* were calculated, and the hierarchical method “complete linkage clustering” was applied to construct the clustering tree, which was displayed using the R package. The expression patterns of the *OsG1*/*G1Ls* were estimated and classified according to the hierarchical clustering results.

### 2.5. Coexpression Analysis with the MADS-Box Gene Family in Rice

All known MADS-box genes were obtained from the TIGR database via a putative function inquiry using the keyword “MADS.” All expression profile data, including those for all of the MADS-box genes, which cover 33 ZS97 and MH63 tissues, were collected from the CREP (http://crep.ncpgr.cn) database. The R language was used to calculate Pearson's correlation coefficients for *OsG1*/*G1Ls* and MADS-box genes, and the “as.dist” function was used to calculate the Euclidean distance matrix. The hierarchical clustering tree of coexpression was constructed with the “hclust” function using the Euclidean distance matrix. The coexpression network was finally constructed by igraph software with a cut-off value of 0.5.

### 2.6. Detection of Expression Profiles of the *OsG1/G1Ls* in Rice

The growth of ZS97 was maintained under routine field management in Wuhan (30°52′N, 114°32′E), China. To be consistent with the samples used in the previous microarray data (see Supplementary Table S1), the samples were harvested from ZS97 plants at the corresponding stages, including seedlings (three-leaf stage, Z12), young shoots (seedlings with two tillers, Z13), young roots (seedlings with 2 tillers, Z14), mature sheaths (secondary branch primordium differentiation stage, Z17), young flag leaves (5 days before heading, Z19), old flag leaves (14 days after heading, Z20), young panicles (4–5 cm, Z24), old panicles (heading stage, Z25), young stems (5 days before heading, Z26), spikelets (3 days after pollination, Z29), and endosperms (7 days after pollination, Z31). Calli (15 days after induction, Z4) were obtained after callus induction using ZS97 seeds. Total RNA was isolated from each sample with the TRIzol reagent (Invitrogen, Carlsbad, CA, USA), according to the manufacturer's instructions. The total RNA was treated with RNase-free DNase I (New England Biolabs, Ipswich, MA, USA) to eliminate genomic DNA contamination. Quantitative real-time PCR (qRT-PCR) was performed with the same procedures as previously reported [11]. The primers used in the present study are listed in Supplementary Table S2.

## 3. Results

### 3.1. Identification of ALOG Domain Genes

To identify ALOG domain genes in rice (*Oryza sativa*) and six other species, including *Physcomitrella patens*, *Brachypodium distachyon*, *Zea mays*, *Sorghum bicolor*, *Arabidopsis thaliana*, and *Populus trichocarpa*, a combined HMMER and BLAST search was performed on a local database as described in Materials and Methods. We finally identify a total of eighty-four sequences containing the ALOG domain from these seven species (see Supplementary Table S3). Specifically, only four ALOG domain genes are identified in *Physcomitrella patens*, while maize possesses nineteen ALOG domain genes in the present study (see Supplementary Table S3). A total of fourteen ALOG domain genes are identified in rice, among which four genes are novel ALOG domain genes compared with previous studies, which are sequentially named *OsG1L10* to *OsG1L13* in the present study [20, 23]. Furthermore, six of the fourteen *OsG1*/*G1Ls* have full-length cDNA in KOME, and only *OsG1L13* is absent in the UniProt annotation result (see Supplementary Table S4). Additionally, probe sets of 11 *OsG1*/*G1Ls* are found in the CREP database, supporting the subsequent expression analysis of ALOG domain genes in rice. Moreover, the number of amino acids of 13 OsG1/G1L proteins varies from 180 to 284, except for *OsG1L12*, with the shortest protein length of only 102 amino acids.

### 3.2. Chromosomal Distribution and Duplication Events of the ALOG Domain Genes in Rice

**Figure 1:**
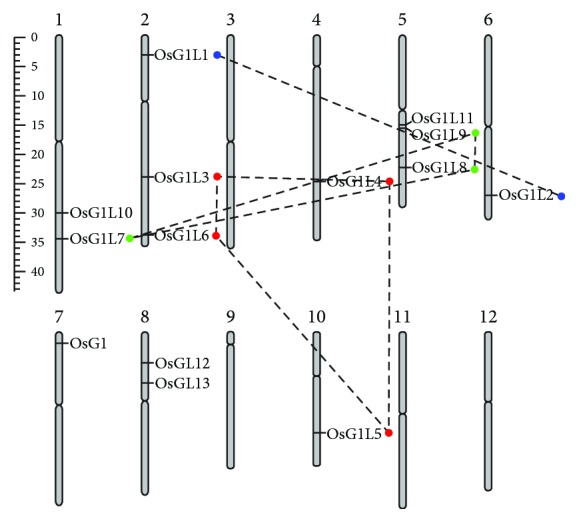
Genomic location and segmental genome duplication of the *OsG1*/*G1Ls*. The chromosome numbers are indicated at the top of each chromosome, and the genes present on the duplicated chromosomal segments of the genome are connected by lines. The scale on the left is in megabases (MB). The segmental duplicated genes were elucidated from PGDD with a parameter of 500 kb.

To show the chromosome location maps of the fourteen *OsG1*/*G1Ls*, the 12 rice chromosomes were drawn as the chromosome pseudomolecules available in TIGR. The result shows that *OsG1*/*G1Ls* are unevenly distributed among eight of the twelve rice chromosomes (Figure 1). Generally, ten *OsG1*/*G1Ls* are located on 4 chromosomes, including two genes on Chr. 1 (*OsG1L7* and *OsG1L10*), three genes on Chr. 2 (*OsG1L1*, *OsG1L3*, and *OsG1L6*), three genes on Chr. 5 (*OsG1L8*, *OsG1L9*, and *OsG1L11*), and two genes on Chr. 8 (*OsG1L12* and *OsG1L13*). By contrast, the other four *OsG1*/*G1Ls*, *OsG1L4*, *OsG1L2*, *OsG1*, and *OsG1L5*, are evenly located on Chr. 4, Chr. 6, Chr. 7, and Chr. 10, respectively. In addition, the *OsG1*/*G1Ls* identified on Chr. 1, Chr. 5, and Chr. 8 tend to locate on the same chromosome arm within each chromosome. However, the *OsG1*/*G1Ls* on Chr. 2 are randomly allocated.

Segmental duplication and tandem duplication play important roles in generating the members of a gene family during evolution [42]. In the present study, three groups of *OsG1*/*G1Ls* are involved in segmental duplication events. In the first group, *OsG1L3*, *OsG1L6*, *OsG1L4*, and *OsG1L5* consist of four putative duplicated gene pairs, including *OsG1L3*/*OsG1L4*, *OsG1L3*/*OsG1L6*, *OsG1L4*/*OsG1L5*, and *OsG1L5*/*OsG1L6*. The second group contains three duplicated gene pairs formed by any two genes of *OsG1L7*, *OsG1L8*, and *OsG1L9*. The third group contains only one duplicated gene pair of *OsG1L1*/*OsG1L2* (Figure 1). However, no tandem duplication is identified among the *OsG1*/*G1Ls*. To assess the duplication time of the segmentally duplicated *OsG1*/*G1Ls*, the synonymous (Ks) and nonsynonymous substitutions (Ka), and the Ka/Ks ratios have been obtained from PGDD. The Ka/Ks ratios of each segmental duplicated gene pair range from 0.28 to 0.79, with an average of 0.53 (smaller than 1), implying that the *OsG1*/*G1Ls* underwent relatively strong purifying selection. The approximate dates of the segmental duplication events were calculated from Ks, and all segmental duplications of the *OsG1*/*G1Ls* occurred 22.04-41.12 million years ago (see Supplementary Table S5). Further analysis of the duplication events in the seven species revealed that no duplication arose between dicots and monocots, and 54 of 57 (94.7%) duplication events occurred no more than 150 million years ago (see Supplementary Table S5). These results suggested that the expansion of the ALOG gene family mainly occurred after the divergence of the monocotyledonous and dicotyledonous plants approximately 200 million years ago [43].

### 3.3. Phylogenetic and Structure Analysis of ALOG Domain Genes

**Figure 2:**
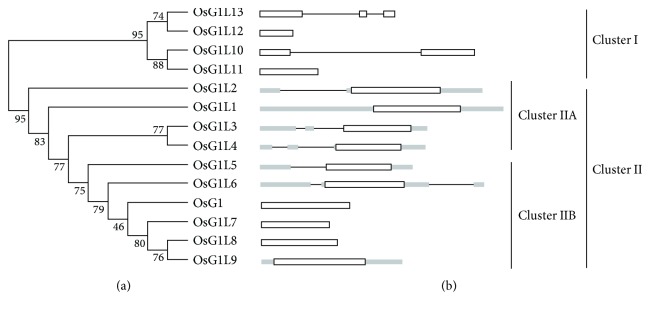
Phylogenetic relationships of the OsG1/G1L proteins and the exon-intron organizations of the corresponding genes. (a) The unrooted tree was generated with the default parameters and the “aLRT” statistical test model using PhyML. (b) Using the sequences from the TIGR locus in Table S4, the exons, introns, and UTRs are represented by white boxes, lines, and black boxes, respectively.

To examine the evolutionary relationships among the *OsG1*/*G1Ls* in rice, an unrooted phylogenetic tree was constructed by PhyML (for details, see Materials and Methods). The 14 *OsG1*/*G1Ls* are classified into two distinct clusters with three subclasses named cluster I, cluster IIA, and cluster IIB (Figure 2(a)). Interestingly, all four newly identified members of the rice ALOG domain gene family, *OsG1L10*, *OsG1L11*, *OsG1L12*, and *OsG1L13*, are included in cluster I. Furthermore, the three genes with known functions, *OsG1*, *OsG1L5*, and *OsG1L6*, are all included in cluster II; however, *OsG1L5* and *OsG1L6* are in the same subclass of cluster IIA, whereas *OsG1* is assigned to the other subclass of cluster IIB. The phylogenetic data also indicate that *OsG1L7*, *OsG1L8*, and *OsG1L9* are paralogs of *OsG1* (cluster IIB) and that *OsG1L3*, *OsG1L4*, and *OsG1L5* are paralogs of *OsG1L6* (cluster IIA). Moreover, *OsG1* and *OsG1L6* are also paralogous genes, which share a similar function in regulating the development of sterile lemma and lemma/palea, respectively [20, 21]. In addition, comparison of the gene structures revealed that all the four genes in cluster I do not have UTRs and that none of the genes in cluster IIB contain introns, but almost all the genes in cluster IIA possess both introns and UTRs (Figure 2(b)). These data demonstrated that the three phylogenetically classified groups in rice share similar gene structures within each group.

**Figure 3:**
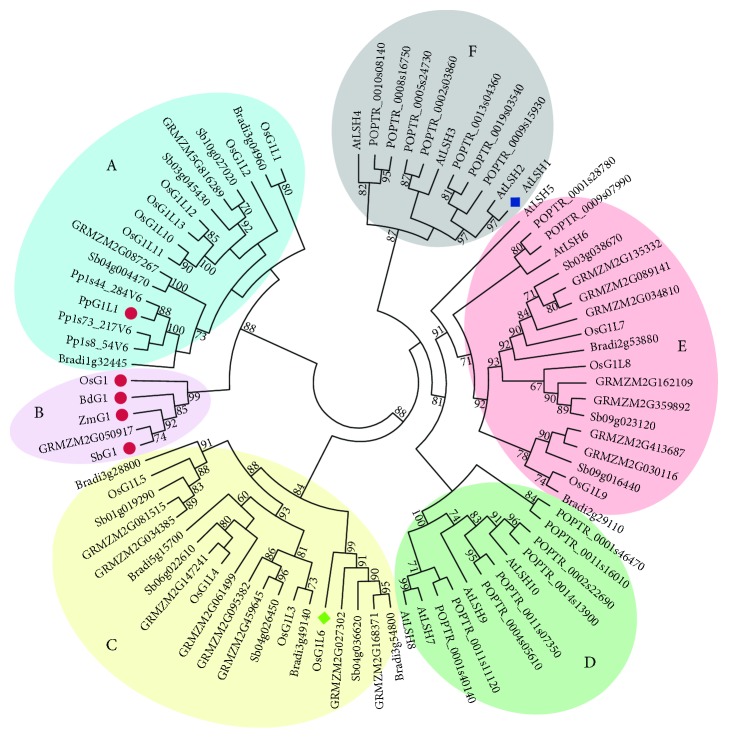
Phylogenetic relationships of the ALOG domain proteins in rice and six other species. Subgroups A to F are indicated in different colored ovals.

To better understand the evolutionary relationships of the ALOG domain genes among the seven species, phylogenetic analysis of all ALOG proteins from the seven species was performed (Figure 3). The phylogenetic tree divides the ALOG domain genes into six distinct clusters (clusters A to F). The three groups of segmental duplicated genes of *OsG1*/*G1Ls* are assigned to clusters A, C, and E. *OsG1*, *BdG1*, *SbG1*, and *ZmG1* are orthologous genes in cluster B, which shows a similar classification with the previously reported G1 clade [20]. Moreover, *GRMZM2G050917* is identified as another ortholog of *OsG1* in maize, indicating that these four orthologs of *OsG1* might have the same function as *OsG1*. Similarly, the data have revealed four orthologs in the other three species for *OsG1L5* (*Bradi3g28800*, *Sb01g019290*, *GRMZM2G081515*, and *GRMZM2G034385*) and *OsG1L6* (*GRMZM2G027302*, *GRMZM2G168371*, *Sb04g036620*, and *Bradi3g54800*). Interestingly, the ALOG domain genes in clusters B, C, and E are all from monocotyledons, and the ALOG domain genes in clusters D and F are all from dicotyledons (Figure 3), suggesting that ALOG domain genes are evolutionarily conserved within monocotyledons or dicotyledons. Although cluster A contains the four genes of *P. patens*, the other thirteen genes are all from monocotyledons, indicating a close evolution of *P. patens* genes with monocotyledons. Taken together, these results provide evidence for the functional research of the uncharacterized but evolutionarily conserved genes.

To gain further insights into the structural diversity of ALOG domain genes, we first compared the exon/intron structure in the coding sequences of ALOG genes (see Supplementary Figure S1). Most of the genes from the dicotyledonous plants seem to lack introns, while the genes from the monocotyledons display more complicated gene structures, and most of them contain introns. Notably, all genes from *B. distachyon* do not contain any intron. However, no uniform structural pattern was observed for the genes in *P. patens*. Thereafter, the MEME motif search tool was used to investigate the conserved motifs of the ALOG protein sequences. We identify five distinct conserved motifs among the ALOG proteins from rice and the other six species (see Supplementary Figure S2). Motifs 1, 2, and 3 specify the ALOG domain, and motifs 4 and 5 are newly discovered in the present study (see Supplementary Table S6). Motifs 1, 2, and 3 are observed in almost all members of the ALOG domain gene family in the seven species, with the exception of the four newly discovered rice genes (*OsG1L10*, *OsG1L11*, *OsG1L12*, and *OsG1L13*) and two maize genes (*GRMZM2G050917* and *GRMZM2G089141*), which lack one or two of the three motifs. This result suggested that motifs 1, 2, and 3 are highly conserved among the seven species. Significantly, although the function of motifs 4 and 5 is unknown, these two motifs are only present in monocotyledonous proteins from cluster C, indicating that motifs 4 and 5 might have specific functions in monocots.

### 3.4. The Tissue-Specific Expression Patterns of *OsG1/G1Ls* Revealed the Potential Biological Functions

**Figure 4:**
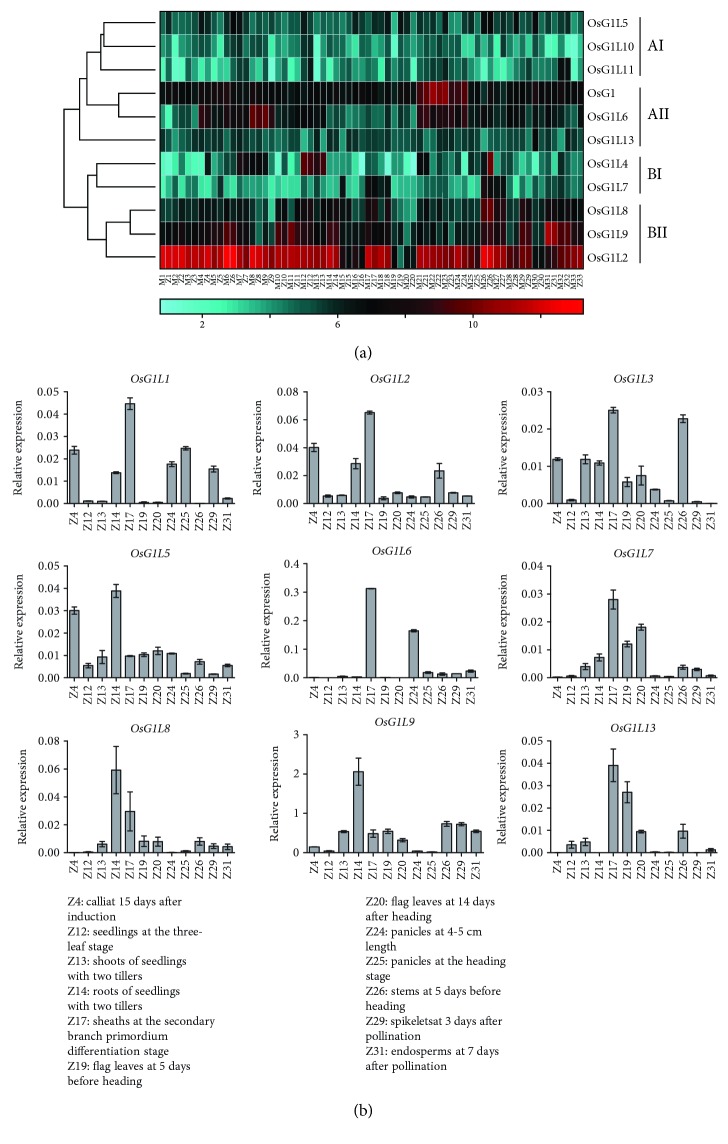
Genome-wide expression profiles and qRT-PCR analysis of *OsG1*/*G1Ls*. (a) The hierarchical clustering displays the expression profiles of the 11 *OsG1*/*G1Ls* using the matching probes in the Affymetrix microarray. The color scale at the left bottom represents the log_2_-transformed expression values. Cyan indicates a low expression level, black indicates a medium expression level, and red indicates a high expression level. The thirty-three samples of MH63 and ZS97 were sequentially marked as M1 to M33 and Z1 to Z33 on the *x*-axis, respectively. The detailed sample information was listed in Supplementary Table S1. (b) qRT-PCR analysis of 9 *OsG1*/*G1Ls*. The twelve analyzed samples were indicated as Z4 to Z31 on the *x*-axis because they were sampled according to the tissues and developmental stages in the microarray (see Supplementary Table S1). The values on the *y*-axis represent the relative expression levels. The error bars represent the standard deviation of the means of three independent replicates.

To explore the expression patterns and potential functions of the *OsG1*/*G1Ls*, we obtained the microarray data for 11 *OsG1*/*G1Ls* in 33 tissues from two cultivated varieties (ZS97 and MH63), which covered almost the entire life cycle of rice. A hierarchical clustering analysis based on the signal values showed that the expression profiles of *OsG1*/*G1Ls* can be classified into two major groups of A and B and further subdivided into four distinct clusters of AI, AII, BI, and BII (Figure 4(a)). *OsG1* and *OsG1L6* exhibit uniquely high expression levels in the panicles, which is spatially consistent with their function in regulating the development of floral organs and panicles [20–22, 25, 44]. Furthermore, *OsG1L6* also displays high expression in the plumules, under both dark and light conditions, implying that *OsG1L6* has other functions besides regulating panicle development. In fact, a previous study has already revealed the pleiotropic effect of *OsG1L6* in regulating plant height, floral development, and grain yield in rice [24]. The present data also showed that the newly discovered *OsG1L10* and *OsG1L11* genes belonging to cluster AI are expressed at extremely low levels in almost all studied tissues. In contrast, *OsG1L2* exhibits remarkably high expression levels in almost every tissue. These results indicated that *OsG1L10* and *OsG1L11* might have lost their functions, whereas *OsG1L2* plays an important role throughout the development of rice and likely represents a housekeeping gene in rice. In addition, *OsG1L4* in cluster BI is highly expressed in seedlings, young shoot, and young stem, while *OsG1L7* in the same cluster has a relatively high expression level in mature sheath and stem (Figure 4(a)). Taken together, these results suggested that the *OsG1*/*G1Ls* have evolved specific biological functions in diverse tissues throughout the rice life cycle.

To further confirm the tissue-specific expression patterns of the *OsG1*/*G1Ls*, the relative expression levels of 9 *OsG1*/*G1Ls* covering the four clusters AI, AII, BI, and BII were investigated in 12 tissues by qRT-PCR (Figure 4(b)). The expression patterns demonstrated several interesting results. First, six genes, including *OsG1L1*, *OsG1L2*, *OsG1L3*, *OsG1L6*, *OsG1L7*, and *OsG1L13*, exhibit high expression levels in mature sheaths (Z17), revealing a potential role for these genes in regulating plant height or the physiological function of rice sheath. Second, the remaining three genes, *OsG1L5*, *OsG1L8*, and *OsG1L9*, are highly expressed in young roots (Z14), and *OsG1L5* also shows high expression levels in calli (Z4). Since *OsG1L5*/*TAWAWA1* was formerly revealed to regulate meristem activity in rice shoot apex [28], the expression of *OsG1L5* in young roots and calli can be explained by the fact that the root apex and calli also contain an abundance of meristems. However, the absence of *OsG1L8* and *OsG1L9* in calli (Z4) suggested that these genes might have a different molecular function than *OsG1L5*. Moreover, the expression patterns of *OsG1L8* and *OsG1L9* revealed by qRT-PCR are highly similar to those revealed by microarray, particularly the relatively high expression levels in Z14, Z17, Z26, and Z29 of both datasets (Figures 4(a) and 4(b)), which experimentally confirmed the reliability of the microarray data. Third, when we compared the expression of *OsG1*/*G1Ls* between young (Z24) and old (Z25) panicles, most genes show similar expression levels. For instance, *OsG1L1* shows similar high expression levels, and *OsG1L2* exhibits equivalent low expression levels, while *OsG1L7*, *OsG1L8*, *OsG1L9*, and *OsG1L13* are simultaneously absent in these two tissues. However, *OsG1L3*, *OsG1L5*, and *OsG1L6* show higher expression levels in young panicle than in old panicle. These data were also supported by the fact that both *OsG1L5* and *OsG1L6* primarily function in young panicles to regulate the development of inflorescence and spikelet [21, 23, 25, 28]; thus, their expression levels can hardly be detected in old panicles.

### 3.5. Coexpression Analysis Reveals the Underlying Relationships between *OsG1/G1Ls* and MADS-Box Genes in Rice

**Figure 5:**
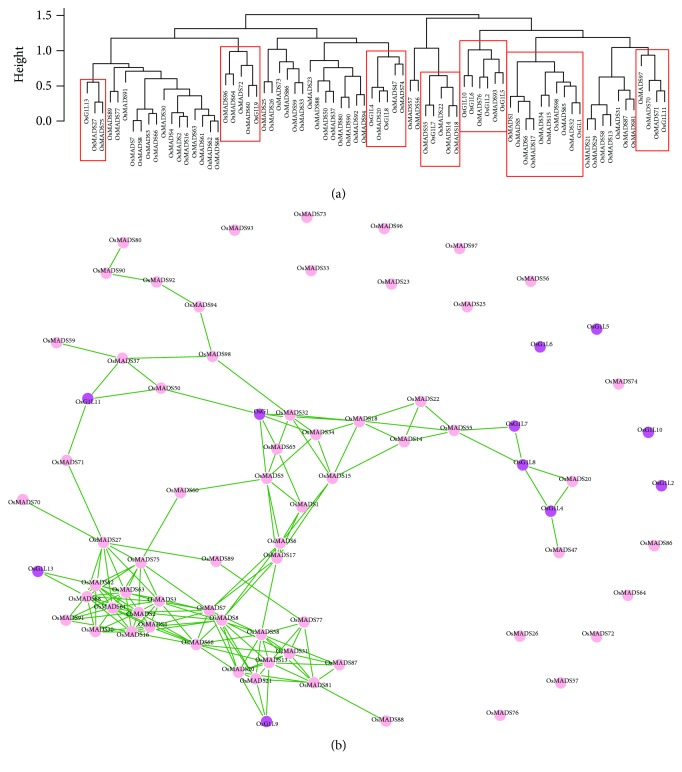
The hierarchical clustering tree and coexpression network between *OsG1/G1Ls* and MADS-box genes in rice. (a) The hierarchical cluster dendrogram showing coexpression modules. The red boxes represent the analyzed coexpression subgroups. The hierarchical clustering tree of coexpression was constructed with the hclust function in R. (b) The gene coexpression network between *OsG1/G1Ls* and *OsMADS* genes. The green lines between two genes represent the coexpression relationships, and their length represents the coexpression signal intensity. The coexpression network was drawn by igraph software with a cut-off value of 0.5.

The previously reported *OsG1*/*G1Ls* mainly participate in the developmental regulation of floral organs in rice [20, 21, 23–25, 28]. It is well known that the identities of the floral organs in plants are controlled by the combined activities of MADS-box genes. To reveal the underlying regulatory relationships of *OsG1*/*G1Ls* and MADS-box genes, we constructed a hierarchical clustering tree and a network of coexpression by using the microarray data of *OsG1*/*G1Ls* and MADS-box genes in rice. The clustering results revealed that *OsG1* is classified into the same cluster with *OsMADS32*, *OsMADS65*, *OsMADS98*, *OsMADS15*, *OsMADS34*, *OsMADS17*, *OsMADS1*, OsMADS6, and *OsMADS5* (Figure 5(a)). Expectedly, *OsMADS1*, *OsMADS17*, and *OsMADS34* are in the same cluster because *OsMADS17* is a direct target gene of *OsMADS1*, and *OsMADS1* can negatively regulate the expression of *OsMADS34* in young spikelets [45, 46]. Coincidentally, the lodicules and stamens are converted to multiple lemma/glume-like organs in the *OsMADS1* mutant, which shows almost the same phenotype as the *OsG1* mutant [20, 45]. These results strongly suggested that *OsMADS1* and *OsG1*, perhaps as well as other genes in this cluster, function in the same molecular pathway to specify floral organ identity. Additionally, stem elongation in the double mutant of *OsMADS22* and *OsMADS55* is markedly reduced [47], and *OsG1L7* is highly coexpressed with these two genes (Figure 5(a)). Moreover, *OsG1L7* shows a relatively high expression level in the sheath and stem (Figures 4(a) and 4(b)), suggesting that *OsG1L7* functions synergistically with *OsMADS22* and *OsMADS55* to regulate stem elongation.

Subsequently, the coexpression network between *OsG1*/*G1Ls* and MADS-box genes was constructed by using igraph (Figure 5(b)). The network shows complicated relationships among different MADS-box genes; however, a much simpler network is detected for *OsG1*/*G1Ls*. For example, *OsG1L4*, *OsG1L7*, *OsG1L8*, *OsG1L9*, *OsG1L11*, and *OsG1L6* show interaction networks with only one to three MADS-box genes. However, *OsG1* was found to play a pivotal role in integrating the interaction between *OsMADS50* and a network composed of many other MADS-box genes, including *OsMADS32*, *OsMADS18*, *OsMADS34*, *OsMADS65*, *OsMADS5*, *OsMADS14*, *OsMADS15*, *OsMADS1*, *OsMADS6*, and *OsMADS17* (Figure 5(b)). Interestingly, *OsMADS50* is an important flowering activator considered to be an upstream regulator of *OsMADS1*, *OsMADS14*, *OsMADS15*, and *OsMADS18* [48]. Taken together, this coexpression network further indicated that *OsG1* functions between *OsMADS50* and the downstream MADS-box genes, such as *OsMADS1*, to regulate the development of inflorescence in rice. However, the authentic relationships between the *OsG1*/*G1Ls* and MADS-box genes need more molecular and genetic evidence, which could be accomplished in future studies.

### 3.6. Comparative Analysis of the Expression Patterns of ALOG Domain Genes in *Arabidopsis* and Maize

**Figure 6:**
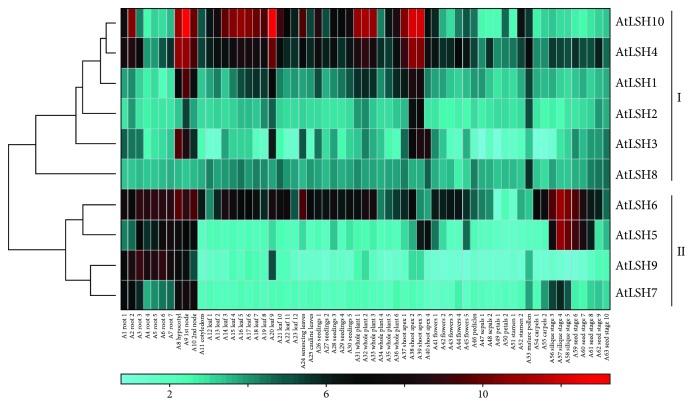
Genome-wide expression profile of ALOG domain genes in *Arabidopsis*. The hierarchical clustering displays the expression profiles of the 10 ALOG domain genes (*AtLSH1* to *AtLSH10*). The color scale at the top left represents the log_2_-transformed expression values. Cyan indicates a low expression level, black indicates a medium expression level, and red indicates a high expression level.

To investigate the potential roles of the ALOG domain genes in *Arabidopsis* and maize, we determined the expression patterns of all ALOG domain genes in these two species by using the available microarray data. The ALOG domain genes in *Arabidopsis* are primarily divided into two major groups (I and II) based on the hierarchical clustering analysis (Figure 6). The first group (I) is represented by *AtLSH1*, *AtLSH3*, *AtLSH4*, and *AtLSH10*, which show high expression levels in the hypocotyl (A8), 1st node (A9), 2nd internode (A10), and shoot apex (A37-A39). These data suggested that these genes regulate the development of the hypocotyl and shoot apex. Indeed, *AtLSH1* mediates a light-dependent regulation of hypocotyl development [26]; *AtLSH3* and *AtLSH4* are important for the maintenance of shoot apical meristem and floral organ development [49, 50]. Furthermore, *AtLSH4* and *AtLSH10* display a strong coexpression pattern and are highly expressed almost throughout the *Arabidopsis* life cycle, suggesting that both genes are functionally important in most of the developmental stages. However, *AtLSH2* exhibits a similar expression pattern with *AtLSH1* but is expressed only in the shoot apex (A38 and A39), suggesting a more specified function of *AtLSH2* in the shoot apex. The second group (II) is composed of *AtLSH5*, *AtLSH6*, *AtLSH7*, and *AtLSH9*, which display high expression levels in the root, hypocotyl, and node (A1-A10). This expression pattern is consistent with the revealed role of *AtLSH9* in the regulation of the hypocotyl length [51] and indicates that *AtLSH5*, *AtLSH6*, *AtLSH7*, and *AtLSH9* might have a similar function in hypocotyls as well as unknown functions in roots. Notably, *AtLSH5*, *AtLSH6*, and *AtLSH7* might also function in the development of siliques since these genes all show obviously high expression levels in siliques (A56-A58), except for *AtLSH9*.

On the other hand, we found that the ALOG domain genes in maize also exhibit tissue-specific expression patterns that were similar to those in rice and *Arabidopsis* (see Supplementary Figure S3). For instance, *GRMZM2G459645*, *GRMZM2G095382*, *GRMZM2G081515*, *GRMZM2G147241*, *GRMZM2G061499*, and *GRMZM2G034385* are highly expressed in the stem and shoot apical meristem (SAM), resembling the highly expressed *AtLSH1*, *AtLSH3*, *AtLSH4*, and *AtLSH10* in Arabidopsis hypocotyl, node, and shoot apex. Furthermore, *GRMZM2G162109*, *GRMZM2G168371*, and GRMZM2G027302 are not only expressed in the stem and SAM but also highly expressed in the husk and seeds. Specifically, *GRMZM2G168371* and *GRMZM2G027302*, the maize orthologs of rice *OsG1L6*, exhibit robustly high expression levels in maize husk and seeds, suggesting that these two maize orthologs might have the same function as *OsG1L6*. This result demonstrated a conserved role of ALOG domain genes in regulating the development of inflorescence in maize and rice. However, the orthologous gene of *OsG1* in maize, *GRMZM2G050917*, shows extremely low expression levels in almost all examined tissues, which implies that this ortholog may have lost its molecular function in maize. Taken together, a comparative analysis of tissue-specific expression patterns of ALOG domain genes has provided an alternative strategy to reveal their conserved biological functions among species.

## 4. Discussion

In the present study, we conducted a comprehensive bioinformatics analysis of ALOG domain genes in rice and six other species. A total of eighty-four ALOG domain genes are identified, among which fourteen *OsG1*/*G1L*s are found to be unevenly distributed on eight of the twelve chromosomes. Phylogenetic and structural analysis showed that ALOG domain genes are evolutionarily conserved within monocotyledons or dicotyledons and that the ALOG domain-specified motifs 1, 2, and 3 are highly conserved among different species. However, the newly identified motifs 4 and 5 are only present in monocots, presenting a specific function of these two motifs in monocots. The expression analysis showed that *OsG1*/*G1Ls* possess specific biological functions in diverse tissues throughout the rice life cycle. The coexpression results revealed that *OsMADS1* and *OsG1* function in the same molecular pathway to specify floral organ identity; *OsG1L7* is potentially associated with *OsMADS22* and *OsMADS55* to regulate stem elongation. Moreover, the coexpression network showed that *OsG1* integrates *OsMADS50* and the downstream MADS-box genes to regulate the development of rice inflorescence. Finally, the comparative expression analysis revealed the conserved biological functions of ALOG family genes among rice, maize, and *Arabidopsis*. These results have shed light on the functional study of ALOG family genes, which will further assist plant breeders for selecting candidate genes during the breeding of rice and other crops.

The present data suggested that segmental duplications mainly contributed to the expansion of *OsG1*/*G1Ls*, which occurred 22.04-41.12 million years ago. However, the average Ka/Ks ratio of the segmental duplicated gene pairs is 0.53, indicating that *OsG1*/*G1Ls* are under purifying selection. Moreover, all Ka/Ks ratios of the duplicated genes within the seven species are less than 1, implying that the ALOG family genes are undergoing strong purifying selection (see Supplementary Table S5). In natural selection, purifying selection or negative selection involves the selective removal of alleles that are deleterious, which could also be applied in artificial selection to preserve preferred traits and remove unwanted traits according to human needs. As previous studies reported, the mutation of ALOG family genes is typically associated with unwanted traits, such as abnormal phenotype of hulls in the rice mutant of *OsG1* and *TH1*/*OsG1L6* [20, 21, 25] and the single-flower primary inflorescence in the tomato mutant of the *TERMINATING FLOWER* gene, which is an ALOG domain gene identified from tomato [52]. These results can partially explain why the ALOG family genes are under purifying selection and suggest that the reserved ALOG family genes play crucial roles during plant development.

The phylogenetic analysis has divided the ALOG family genes into six clusters, in which monocotyledonous ALOG genes constitute clusters A, B, C, and E, while dicotyledonous ALOG genes are all classified into clusters D and F (Figure 3). The phylogenetic classification seems to be consistent with the gene structure and protein motif distribution. For example, the ALOG family genes of monocotyledonous plants generally contain a number of introns, whereas dicotyledonous plants have fewer introns than the monocots (see Supplementary Figure S1). Furthermore, all of the ALOG family proteins of the dicotyledons do not have putative motifs 4 and 5, which are identified only in monocotyledons (see Supplementary Figure S2). These data not only suggested that the ALOG family genes are evolutionarily and structurally conserved within monocotyledons or dicotyledons but also implied that the ALOG family genes in dicotyledons preferentially lose introns compared with monocotyledons during evolution. This observation was similar to a previous result showing that intron losses are roughly 12.6 and 9.8 times more frequent than intron gains in the recent evolution of *Arabidopsis thaliana* and *Oryza sativa*, respectively [53]. However, there is an exception that all 9 ALOG family genes in *Brachypodium distachyon* contain no introns (see Supplementary Figure S1), indicating that intron losses are occasionally species-specific events. To further reveal the underlying mechanism of structure variation, why and how the intron losses of ALOG family genes, including precise and imprecise intron losses [54], were fixed during evolution should be answered in the future.

*OsG1*/*G1Ls* are composed of fourteen members that were identified in the present study. Currently, several studies have revealed the functional roles of three members in *OsG1*/*G1Ls*, showing that *OsG1* specifies sterile lemma identity in rice spikelets [20], *OsG1L5*/*TAWAWA1* regulates inflorescence development by modifying meristem activity [28], and *OsG1L6*/*TH1*/*BH1*/*AFD1* pleiotropically affects plant height, grain size, and particularly the development of spikelets, lemmas, and paleae [21–25, 44]. Compared to the ALOG gene family in rice, the MADS-box gene family is a much more extended family containing as many as 75 MADS-box genes [55]. Numerous studies have confirmed the importance of MADS-box genes in floral organ specification, plant growth, and resistance to biotic or abiotic stresses [45–48, 56–58]. The overlapping functions of *OsG1*/*G1Ls* and MADS-box genes, particularly their effects on floral organ identity and meristem activity, have prompted us to investigate their relationships by coexpression analysis. The results showed that *OsG1* probably functions between *OsMADS50* and many other MADS-box genes, such as *OsMADS32*, *OsMADS18*, *OsMADS34*, *OsMADS65*, *OsMADS5*, *OsMADS14*, *OsMADS15*, *OsMADS1*, *OsMADS6*, and *OsMADS17*, to specify floral organ identity. Among these MADS-box genes, *OsMADS32* regulates floral organ identity in rice, *OsMADS1* plays an important role in rice floral organ identity specification and floral meristem determinacy [45, 46, 59, 60], *OsMADS1* and *OsMADS15* are both required to ensure sexual reproduction in rice [61], and *OsMADS34* specifies the identities of floral organs [62, 63]. Based on these results, we propose that *OsG1* functions synergistically with or upstream of *OsMADS1*, *OsMADS15*, *OsMADS32*, and *OsMADS34* genes to specify the identities of floral organs in rice.

Comparative expression analysis within or between species is an efficient method to reveal the potential function of candidate genes. In the present study, we compared the tissue-specific expression patterns of ALOG domain genes among rice, maize, and *Arabidopsis* and found that several ALOG domain genes from different species prefer to express in similar tissues. For example, *AtLSH5*, *AtLSH6*, *AtLSH7*, and *AtLSH9* display high expression levels in the hypocotyl and node, which correspond to the high expression of *OsG1L7* and *OsG1L8* in rice sheath and stem (Figures 4(a) and 6). The high expression of *GRMZM2G162109*, *GRMZM2G168371*, and GRMZM2G027302 in maize husk and seeds resembles the markedly high expression of *OsG1* and *OsG1L6* in rice panicles (Figure 4(a) and Figure S3). The conserved tissue-specific expression patterns of ALOG family genes between species provided the opportunity to investigate their conserved functions. Recently, a high-resolution spatiotemporal transcriptome analysis was successfully applied to reveal the regulatory and structural gene networks across tissue and developmental spectra in tomato [64]. Moreover, a previous time-course transcriptome analysis revealed that the flowering pathway genes are expressed during the same time quantum at approximately 8:00 am, indicating that the temporal consistency of gene expression is necessary for genes in the same pathway [65]. Therefore, future dissection of gene functions or regulatory networks needs more precise expression data from a series of high-resolution spatiotemporal samples.

## Data Availability

The data used to support the findings of this study are available from the corresponding author upon request.

## Supplementary Materials

Supplementary Materials Figure S1: the gene structure of ALOG domain genes in seven species. The size of each gene can be estimated using the black bar (100 bp) at the bottom of the figure. White boxes, exons; gray boxes, UTRs; lines, introns. Figure S2: distribution of conserved motifs in ALOG domain proteins identified using the MEME search tool. Five putative motifs were identified, in which motifs 1, 2, and 3 specify the ALOG domain, while motifs 4 and 5 are newly discovered in the present study. Different motifs are indicated by different colors, and the names of all members are shown at the top of the figure. The motif sizes are indicated at the bottom of the figure. Figure S3: genome-wide expression profile of ALOG domain genes in maize. The hierarchical clustering displays the expression profiles of the 16 ALOG domain genes in maize. The color scale at the top left represents the log_2_-transformed expression values. Cyan indicates a low expression level, black indicates a medium expression level, and red indicates a high expression level. Table S1: tissue samples of different developmental stages throughout the life cycle of rice. Table S2: the qRT-PCR primers used in the present study. Table S3: the eighty-four ALOG domain genes identified from the seven species. Table S4: information and characteristics of the identified OsG1/G1Ls in the rice genome. Table S5: the segmental duplication events of the ALOG family genes. Table S6: the conserved motifs identified from ALOG family proteins.
