# Supplementary material for: Genome-Wide Identification and Characterization of the ALOG Domain Genes in Rice
Source: Int J Genomics. 2019 Feb 24;2019:2146391. doi: 10.1155/2019/2146391 (PMC6409076; doi:10.1155/2019/2146391)
Supplement: Supplementary Materials — Figure S1: the gene structure of ALOG domain genes in seven species. The size of each gene can be estimated using the black bar (100 bp) at the bottom of the figure. White boxes, exons; gray boxes, UTRs; lines, introns. Figure S2: distribution of conserved motifs in ALOG domain proteins identified using the MEME search tool. Five putative motifs were identified, in which motifs 1, 2, and 3 specify the ALOG domain, while motifs 4 and 5 are newly discovered in the present study. Different motifs are indicated by different colors, and the names of all members are shown at the top of the figure. The motif sizes are indicated at the bottom of the figure. Figure S3: genome-wide expression profile of ALOG domain genes in maize. The hierarchical clustering displays the expression profiles of the 16 ALOG domain genes in maize. The color scale at the top left represents the log2-transformed expression values. Cyan indicates a low expression level, black indicates a medium expression level, and red indicates a high expression level. Table S1: tissue samples of different developmental stages throughout the life cycle of rice. Table S2: the qRT-PCR primers used in the present study. Table S3: the eighty-four ALOG domain genes identified from the seven species. Table S4: information and characteristics of the identified OsG1/G1Ls in the rice genome. Table S5: the segmental duplication events of the ALOG family genes. Table S6: the conserved motifs identified from ALOG family proteins. [file 2146391.f1.pdf]

Supplementary Materials for “Genome-wide identification and characterization of the ALOG domain gene family in rice”

Na Li[1], Yang Wang[1], Jing Lu[2] and Chuan Liu[1,3]

[1]State Key Laboratory of Hybrid Rice, College of Life Sciences, Wuhan University, Wuhan 430072, China

[2]College of Food Science and Technology, Huazhong Agricultural University, Wuhan 430070, China

[3]Chongqing Key Laboratory on Big Data for Bio Intelligence, Chongqing University of Posts and Telecommunications, Chongqing 400065, China.

Correspondence should be addressed to Chuan Liu; liuchuan@whu.edu.cn

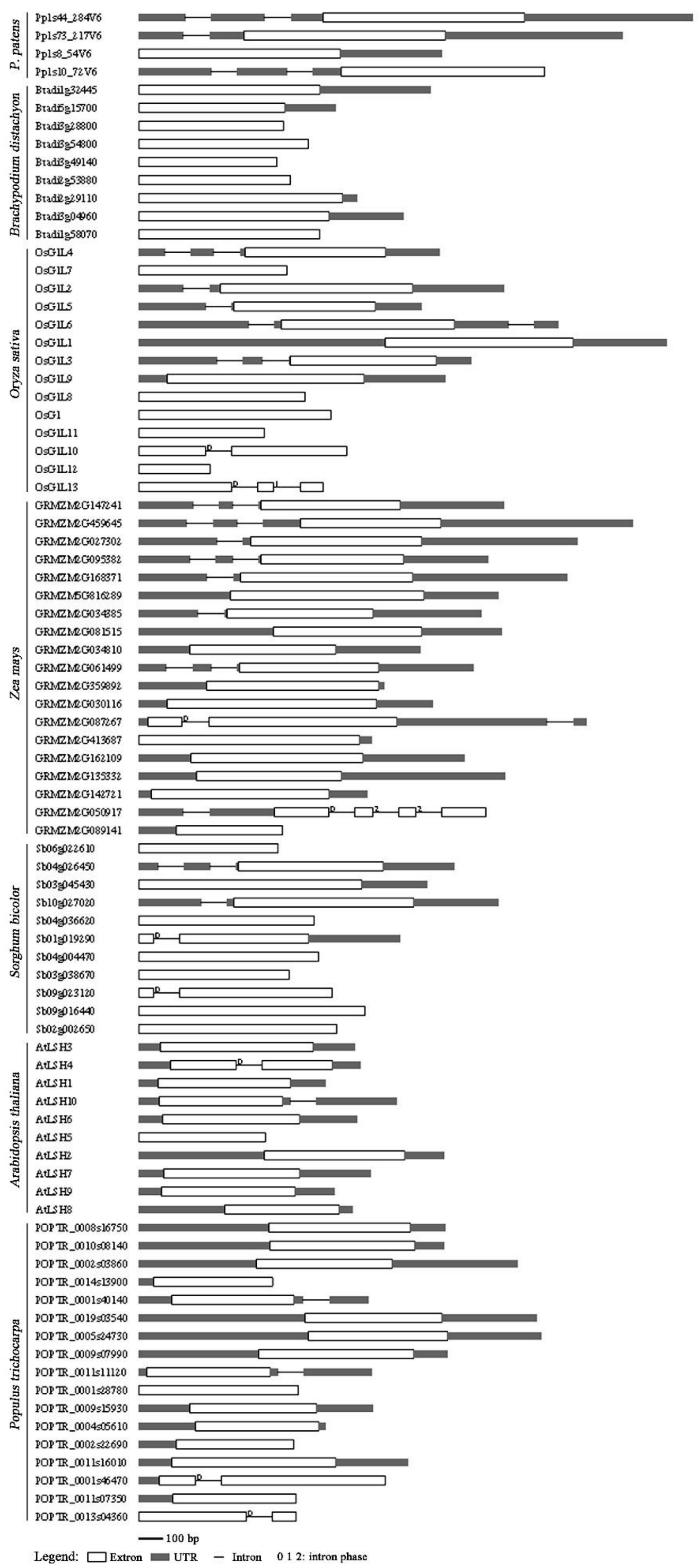

Figure S1: The gene structure of ALOG domain genes in seven species. The size of each gene can be estimated using the black bar (100 bp) at the bottom of the figure. White boxes, exons; gray boxes, UTRs; lines, introns.

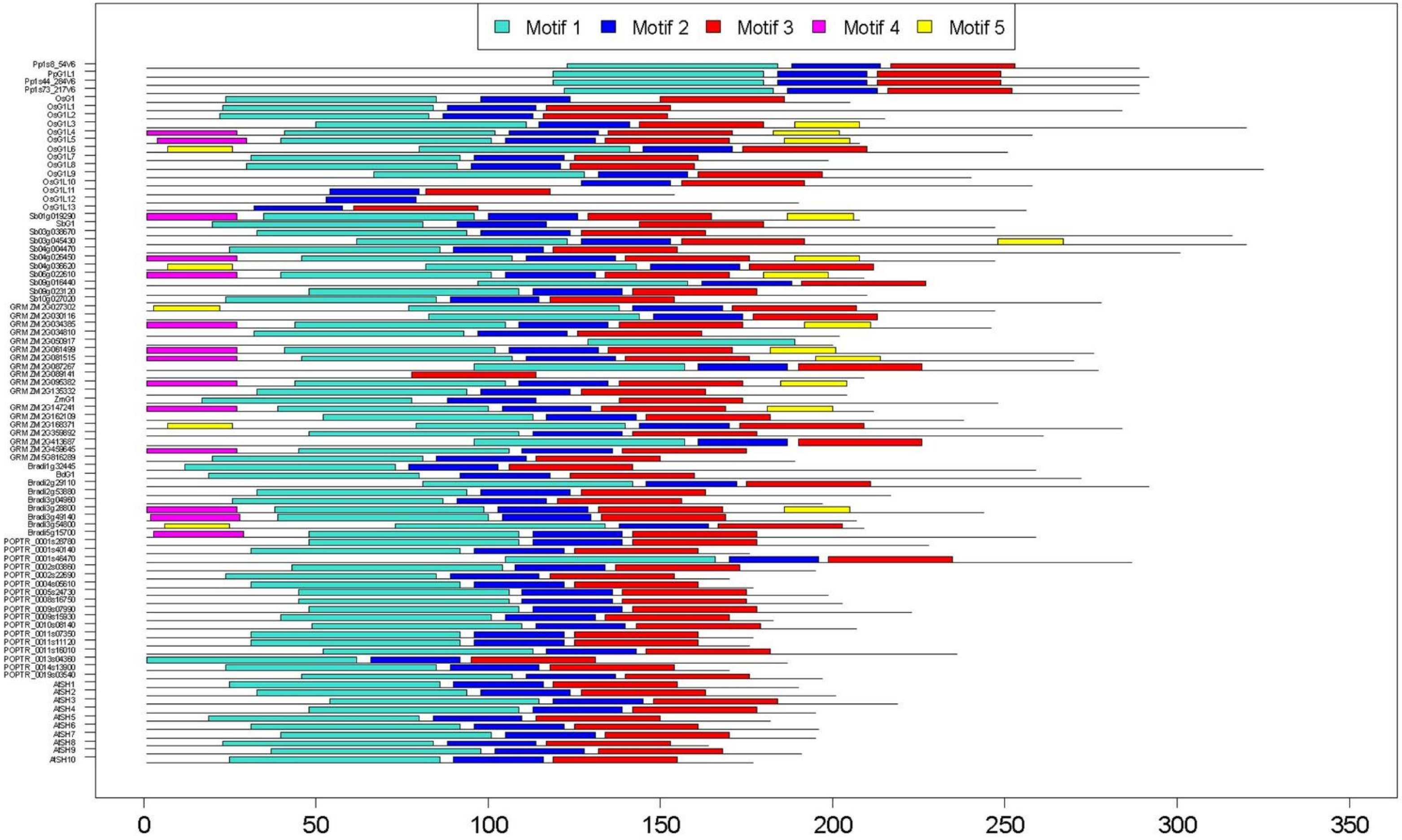

Figure S2: Distribution of conserved motifs in ALOG domain proteins identified using the MEME search tool. Five putative motifs were identified, in which motifs 1, 2 and 3 specify the ALOG domain, while motifs 4 and 5 are newly discovered in the present study. Different motifs are indicated by different colors, and the names of all members are shown at the top of the figure. The motif sizes are indicated at the bottom of the figure.

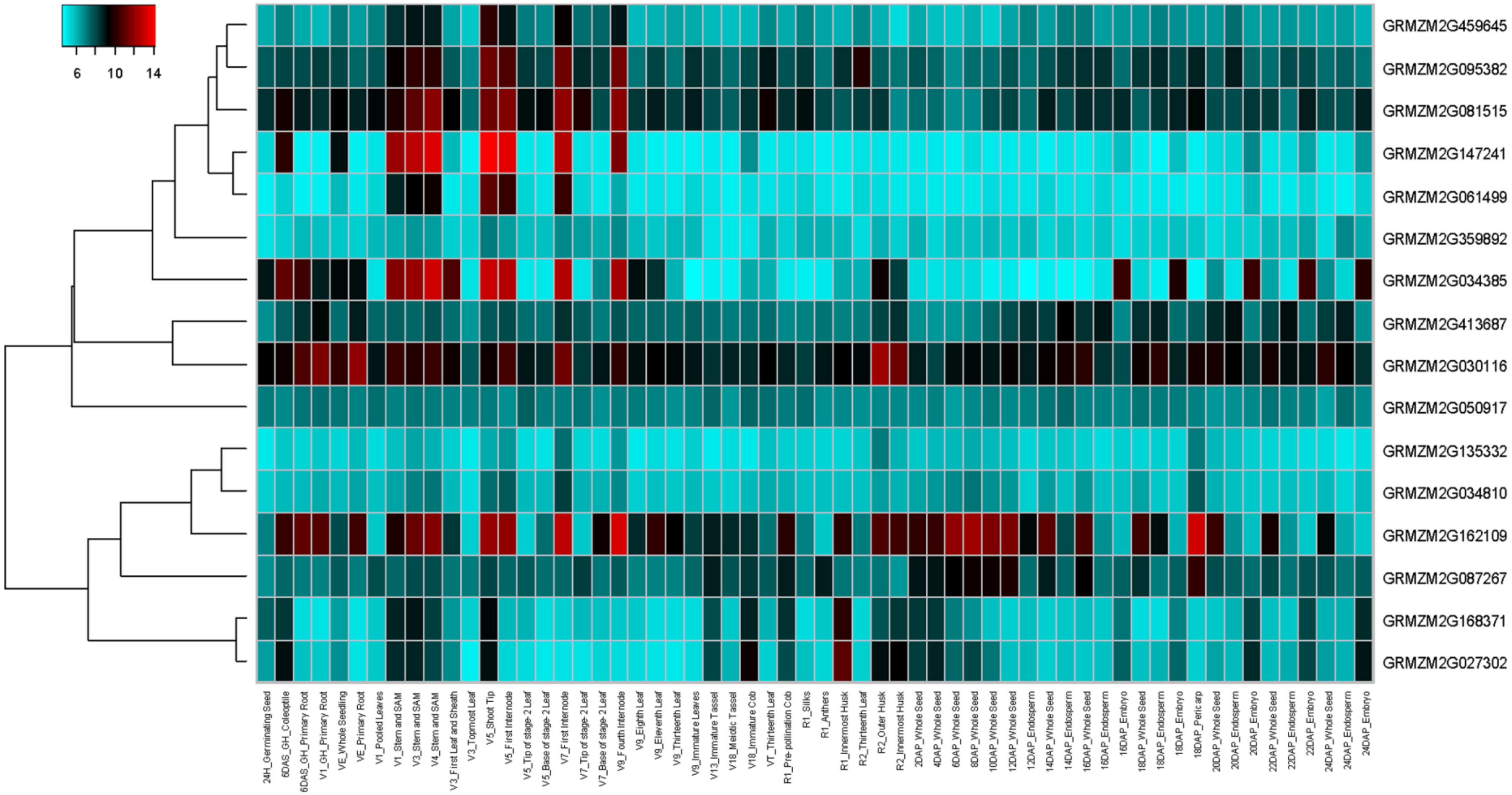

Figure S3: Genome-wide expression profile of ALOG domain genes in maize. The hierarchical clustering displays the expression profiles of the 16 ALOG domain genes in maize. The color scale at the top left represents the log_2-transformed expression values. Cyan indicates a low expression level; black indicates a medium expression level; and red indicates a high expression level.

Table S1: Tissue samples of different developmental stages throughout the life cycle of rice

Sample No	Abbreviation	Tissues or organ	Developmental stages
M1(Minghui)/Z1(Zhenshan)	Calli 1	Calli	15 DAS (days after subculture)
M2(Minghui)/Z2(Zhenshan)	Calli 2	Calli	5 DAR (days after regeneration)
M3(Minghui)/Z3(Zhenshan)	Calli 3	Calli	Screening stage
M4(Minghui)/Z4(Zhenshan)	Calli 4	Calli	15 DAI T2 (days after induction)
M5(Minghui)/Z5(Zhenshan)	Calli 5	Calli	15 DAI T3 (days after induction)
M6(Minghui)/Z6(Zhenshan)	Seed imbibition	Seed	72 h after imbibition
M7(Minghui)/Z7(Zhenshan)	Seed germination	Embryo bud and radicle	3 days after germination
M8(Minghui)/Z8(Zhenshan)	Plumule/Dark	Plumule	48 hours after emergence, dark
M9(Minghui)/Z9(Zhenshan)	Plumule/Light	Plumule	48 hours after emergence, light
M10(Minghui)/Z10(Zhenshan)	Radicle/Dark	Radicle	48 hours after emergence, dark
M11(Minghui)/Z11(Zhenshan)	Radicle/Light	Radicle	48 hours after emergence, light
M12(Minghui)/Z12(Zhenshan)	Seedlings	Seedling	Three-leaf stage
M13(Minghui)/Z13(Zhenshan)	Young shoot	Shoot	Seedlings with 2 tillers
M14(Minghui)/Z14(Zhenshan)	Young root	Root	Seedlings with 2 tillers
M15(Minghui)/Z15(Zhenshan)	Mature leaf	Leaf	Stage 3 (secondary branch primordium differentiation stage)
M16(Minghui)/Z16(Zhenshan)	Old leaf	Leaf	4–5 cm young panicle
M17(Minghui)/Z17(Zhenshan)	Mature sheath	Sheath	Stage 3
M18(Minghui)/Z18(Zhenshan)	Old sheath	Sheath	4–5 cm young panicle
M19(Minghui)/Z19(Zhenshan)	Young flag leaf	Flag leaf	5 DBH (5 days before heading)
M20(Minghui)/Z20(Zhenshan)	Old flag leaf	Flag leaf	14 DAH (14 days after heading)
M21(Minghui)/Z21(Zhenshan)	Panicle, stage 3	Young panicle	Stage 3
M22(Minghui)/Z22(Zhenshan)	Panicle, stage 4	Young panicle	Stage 4 (pistil/stamen primordium differentiation stage)
M23(Minghui)/Z23(Zhenshan)	Panicle, stage 5	Young panicle	Stage 5 (pollen-mother cell formation stage)

Table S1: Continued

Sample No	Abbreviation	Tissues or organ	Developmental stages
M24(Minghui)/Z24(Zhenshan)	Young panicle	Panicle	4–5 cm young panicle
M25(Minghui)/Z25(Zhenshan)	Old panicle	Panicle	Heading stage
M26(Minghui)/Z26(Zhenshan)	Young stem	Stem	5 DBH
M27(Minghui)/Z27(Zhenshan)	Old stem	Stem	Heading stage
M28(Minghui)/Z28(Zhenshan)	Hull	Hull	1 DBF (1 day before flowering)
M29(Minghui)/Z29(Zhenshan)	Spikelet	Spikelet	3 DAP (3 days after pollination)
M30(Minghui)/Z30(Zhenshan)	Stamen	Stamen	1 DBF
M31(Minghui)/Z31(Zhenshan)	Endosperm 1	Endosperm	7 DAP (7 days after pollination)
M32(Minghui)/Z32(Zhenshan)	Endosperm 2	Endosperm	14 DAP (14 days after pollination)
M33(Minghui)/Z33(Zhenshan)	Endosperm 3	Endosperm	21 DAP (21 days after pollination)

Table S2:. The qRT-PCR primers used in the present study

Gene name	Forward primer (5'-3')	Reverse primer (5'-3')
OsG1L1	GCCACTGTCGGTGTTCAACTAG	ATCCCAAAGCAAATGCAAATCA
OsG1L2	CCACCCTACTCCTTCTAGTACCTC	CAGCAAACCTTTCTCCCTCCT
OsG1L3	GGAGAAGAAGAACCAGCAGTAGCA	AGCGGCGGAGAAGAACAAGA
OsG1L5	CACGTCACATCACCTTCCACCTC	ACGACGACGACGACGACCAT
OsG1L6	CTGCTGCCTTGCTTGCTGTCA	CATGCACGAGCACGAGACGAG
OsG1L7	AGCCGTCCGGCTCGTCTTCT	AGTGCCACTCCCTCCAGGTTGA
OsG1L8	ATGGAGGGAGGAGGAGGTGG	CTTCTGCGACTCGTACCTGCTCA
OsG1L9	GCGACGAGTTCGTGATAAGTGT	GGATTAGAATTTGGAGGGTGATG
OsG1L13	GACTGTGCCTGATTCCTTAATGGT	CAGATGGGTTCCAGTTGAAGGT
Actin	TGTATGCCAGTGGTCGTACCA	CCAGCAAGGTCGAGACGAA

Table S3: The eighty-four ALOG domain genes identified from the seven species

Species	Accession	Gene name	Length	Score	E-value
Physcomitrella patens (Moss)	Pp1s44_284V6.1		289	206.2	1.20E-59
	Pp1s73_217V6.1		289	204.9	2.90E-59
	Pp1s8_54V6.1		289	204.9	2.80E-59
	Pp1s10_72V6.1		292	203.8	6.50E-59
Brachypodium distachyon (Purple false brome)	Bradi1g32445.1		259	219.7	7.80E-64
	Bradi5g15700.1		209	219.7	7.60E-64
	Bradi3g28800.1		207	219.6	8.50E-64
	Bradi3g54800.1		244	219.6	8.30E-64
	Bradi3g49140.1		197	218.7	1.60E-63
	Bradi2g53880.1		217	218.2	2.30E-63
	Bradi2g29110.1		292	215.4	1.70E-62
	Bradi3g04960.1		272	214.5	3.10E-62
	Bradi1g58070.1	BdG1	259	160	2.10E-45
Oryza sativa (Rice)	LOC_Os04g43580.1	OsG1L4	202	220.2	5.50E-64
	LOC_Os01g61310.1	OsG1L7	212	220.1	5.80E-64
	LOC_Os06g46030.1	OsG1L2	277	219.6	8.20E-64
	LOC_Os10g33780.1	OsG1L5 (TAWAWA1)	204	219.6	8.40E-64
	LOC_Os02g56610.1	OsG1L6 (TH1/BH1/AFD1)	248	219.2	1.10E-63
	LOC_Os02g07030.1	OsG1L1	270	217.1	5.00E-63
	LOC_Os02g41460.1	OsG1L3	209	217.1	4.90E-63
	LOC_Os05g28040.1	OsG1L9	284	217.1	4.80E-63
	LOC_Os05g39500.1	OsG1L8	238	215.2	1.90E-62
	LOC_Os07g04670.1	OsG1	276	160.5	1.50E-45
	LOC_Os05g27120.1	OsG1L11*	180	121.5	1.70E-33
	LOC_Os01g54180.1	OsG1L10*	261	115.4	1.40E-31
	LOC_Os08g09660.1	OsG1L12*	102	112.9	7.60E-31
	LOC_Os08g14970.1	OsG1L13*	189	102.6	1.20E-27

Table S3: Continued

Species	Accession	Gene name	Length	Score	E-value
	GRMZM2G147241		200	220.5	4.50E-64
	GRMZM2G459645		202	219.7	8.00E-64
	GRMZM2G027302		246	219.6	8.40E-64
	GRMZM2G095382		204	219.6	8.50E-64
	GRMZM2G168371		247	219.2	1.10E-63
	GRMZM5G816289		278	218.9	1.40E-63
	GRMZM2G034385		210	218.6	1.60E-63
	GRMZM2G081515		213	218.1	2.40E-63
	GRMZM2G034810		209	217.8	3.10E-63
Zea mays (maize)	GRMZM2G061499		201	217.4	4.00E-63
	GRMZM2G359892		247	217.2	4.60E-63
	GRMZM2G030116		301	217	5.40E-63
	GRMZM2G087267		320	216.6	6.90E-63
	GRMZM2G413687		316	214.7	2.80E-62
	GRMZM2G162109		247	214	4.60E-62
	GRMZM2G135332		208	207.2	5.80E-60
	GRMZM2G142721	ZmG1	256	158.6	5.80E-45
	GRMZM2G050917		190	50.2	1.90E-11
	GRMZM2G089141		154	39.6	3.40E-08
	Sb06g022610.1		199	220.8	3.60E-64
	Sb04g026450.1		208	220.4	4.90E-64
	Sb03g045430.1		320	219.4	9.40E-64
	Sb10g027020.1		258	219.2	1.10E-63
	Sb04g036620.1		251	219.1	1.20E-63
Sorghum bicolor (Cereal grass)	Sb01g019290.1		205	218.4	2.00E-63
	Sb04g004470.1		258	218.3	2.10E-63
	Sb03g038670.1		215	216.9	5.80E-63
	Sb09g023120.1		240	216.4	7.90E-63
	Sb09g016440.1		325	214.7	2.80E-62
	Sb02g002650.1	SbG1	284	158.4	6.80E-45
	AT2G31160.1	AtLSH3	219	221.9	1.70E-64
	AT3G23290.2	AtLSH4	195	218.9	1.40E-63
	AT5G28490.1	AtLSH1	190	218.4	2.00E-63
	AT2G42610.1	AtLSH10	177	216.4	8.20E-63
	AT1G07090.1	AtLSH6	196	215.6	1.40E-62
Arabidopsis thaliana (Thale cress)	AT5G58500.1	AtLSH5	182	212.8	1.00E-61
	AT3G04510.1	AtLSH2	201	210.7	4.80E-61
	AT1G78815.1	AtLSH7	195	209.9	8.10E-61
	AT4G18610.1	AtLSH9	191	206.9	7.00E-60
	AT1G16910.1	AtLSH8	164	202.7	1.30E-58

Table S3: Continued

Species	Accession	Gene name	Length	Score	E-value
	POPTR_0008s16750.1		203	223.2	6.60E-65
	POPTR_0010s08140.1		207	222.7	9.20E-65
	POPTR_0002s03860.1		195	221.5	2.20E-64
	POPTR_0014s13900.1		170	220.5	4.40E-64
	POPTR_0001s40140.1		176	219.4	9.50E-64
	POPTR_0019s03540.1		197	219	1.20E-63
	POPTR_0005s24730.1		199	218.8	1.50E-63
	POPTR_0009s07990.1		223	218.7	1.60E-63
Populus trichocarpa (Western poplar)	POPTR_0011s11120.1		176	217.1	4.90E-63
	POPTR_0001s28780.1		228	216.7	6.80E-63
	POPTR_0009s15930.1		183	215.3	1.80E-62
	POPTR_0004s05610.1		177	214	4.60E-62
	POPTR_0002s22690.1		170	213.5	6.40E-62
	POPTR_0011s16010.1		236	212.5	1.30E-61
	POPTR_0001s46470.1		287	211.3	3.20E-61
	POPTR_0011s07350.1		177	210.9	4.10E-61
	POPTR_0013s04360.1		187	210.8	4.40E-61

Table S4: Information and characteristics of the identified *OsG1/G1Ls* in the rice genome

TIGR Loci	Gene Name	E-value[a]	NCBI	RAP Locus	Interpro	PFam	UniProt	Probset ID	Protein length
Os07g04670	*OsG1*[b]	1.50E-45	-	Os07g0139300	IPR006936	PF04852	Q652I1	OsAffx.28268.1.S1_at	276
Os02g07030	*OsG1L1*	5.00E-63	AK106854	Os02g0166800	IPR006936	PF04852	Q0E3M2	N/A[c]	270
Os06g46030	*OsG1L2*	8.20E-64	AK068794	Os06g0672400	IPR006936	PF04852	Q6ATW6	Os.52694.1.S1_at	277
Os02g41460	*OsG1L3*	4.90E-63	-	Os02g0623400	IPR006936	PF04852	Q0DZF3	N/A	209
Os04g43580	*OsG1L4*	5.50E-64	AK243311	Os04g0516200	IPR006936	PF04852	Q6K5X1	OsAffx.26447.3.S1_at	202
Os10g33780	*OsG1L5*[b]	8.40E-64	AK243306	Os10g0478000	IPR006936	PF04852	Q7XDD0	OsAffx.26447.1.A1_x_at	204
Os02g56610	*OsG1L6*[b]	1.10E-63	AK111446	Os02g0811000	IPR006936	PF04852	Q6K1Q1	Os.57394.1.S1_at	248
Os01g61310	*OsG1L7*	5.80E-64	-	Os01g0828900	IPR006936	PF04852	Q941W1	OsAffx.23881.1.S1_x_at	212
Os05g39500	*OsG1L8*	1.90E-62	-	Os05g0472000	IPR006936	PF04852	Q5W659	OsAffx.27237.1.S1_x_at	238
Os05g28040	*OsG1L9*	4.80E-63	AK106384	Os05g0347400	IPR006936	PF04852	A3AVK3	Os.54655.1.S1_at	284
Os01g54180	*OsG1L10**	1.40E-31	-	Os01g0745200	IPR006936	PF04852	B9EU95	OsAffx.21629.1.S1_x_at	261
Os05g27120	*OsG1L11**	1.70E-33	-	Os05g0337600	IPR006936	PF04852	Q7XRS1	N/A	180
Os08g09660	*OsG1L12**	7.60E-31	-	Os08g0196000	IPR006936	PF04852	Q8GVZ6	OsAffx.16924.1.S1_x_at	102
Os08g14970	*OsG1L13**	1.20E-27	-	Os08g0248000	IPR006936	PF04852	N/A	OsAffx.29211.1.S1_at	189

a: E-value of HMMER prediction; b: Genes with reported functions; c: Not found;* Gene named in the present study

Table S5: The segmental duplication events of the ALOG family genes

Gene1	Gene2	Ka	Ks	Ka/Ks	Date(million years)	E-value
AT3G04510	AT5G28490	0.09	1.03	0.09	78.97	2.0E-85
AT2G31160	AT2G42610	0.37	1.35	0.27	103.87	5.0E-64
AT1G07090	AT5G58500	0.23	1.90	0.12	146.49	5.0E-84
AT1G16910	AT1G78815	0.16	0.68	0.24	52.00	7.0E-76
AT5G28490	POPTR_0013s04360	0.00	0.00	0.00	0.00	1.0E-73
AT3G04510	POPTR_0019s03540	0.28	3.94	0.07	303.18	2.0E-74
AT3G04510	POPTR_0013s04360	0.27	2.76	0.10	212.09	5.0E-70
AT3G23290	POPTR_0002s03860	0.21	4.40	0.05	338.44	9.0E-76
Bradi2g53880	GRMZM2G162109	0.21	0.70	0.30	53.68	5.0E-78
Bradi2g22561	GRMZM2G162109	0.28	0.63	0.45	48.49	1.0E-76
Bradi2g22561	GRMZM2G135332	0.20	0.77	0.26	59.18	2.0E-78
Bradi2g53880	GRMZM2G135332	0.20	0.73	0.27	56.41	4.0E-81
Bradi2g53880	GRMZM2G359892	0.21	0.63	0.34	48.84	2.0E-80
Bradi2g22561	GRMZM2G359892	0.28	0.74	0.38	57.20	6.0E-76
Bradi2g29110	GRMZM2G030116	0.20	1.03	0.20	79.03	5.0E-75
Bradi1g58070	GRMZM2G142721	0.37	0.64	0.58	49.15	2.0E-53
Bradi3g54800	Bradi5g15700	0.29	0.44	0.66	34.12	6.0E-71
Bradi3g28800	Bradi5g15700	0.18	0.33	0.54	25.24	1.0E-68
Bradi3g49140	Bradi5g15700	0.20	0.44	0.45	33.87	3.0E-71
Bradi3g49140	Bradi3g54800	0.20	0.61	0.32	47.18	4.0E-74
Bradi3g28800	Bradi3g54800	0.27	0.56	0.48	42.70	2.0E-75
Bradi3g28800	Bradi3g49140	0.21	0.39	0.55	29.63	4.0E-71
Bradi2g29110	Bradi2g53880	0.26	0.94	0.28	72.16	1.0E-80
Bradi2g22561	Bradi2g53880	0.20	0.74	0.27	57.30	8.0E-78
Bradi1g32445	Bradi3g04960	0.31	0.53	0.58	40.64	3.0E-72
LOC_Os01g61310	LOC_Os05g28040	0.22	0.53	0.42	41.12	1.0E-77
LOC_Os01g61310	LOC_Os05g39500	0.15	0.45	0.33	34.42	3.0E-83
LOC_Os02g41460	LOC_Os10g33780	0.19	0.47	0.40	36.52	3.0E-69
LOC_Os02g41460	LOC_Os02g56610	0.26	0.44	0.59	33.81	5.0E-74
LOC_Os02g41460	LOC_Os04g43580	0.11	0.41	0.28	31.30	5.0E-79
LOC_Os02g07030	LOC_Os06g46030	0.28	0.36	0.79	27.68	5.0E-74
LOC_Os04g43580	LOC_Os10g33780	0.17	0.29	0.59	22.04	1.0E-69
LOC_Os05g28040	LOC_Os05g39500	0.31	0.50	0.62	38.73	2.0E-76
LOC_Os07g04670	Sb02g002650	0.33	0.44	0.75	34.22	3.0E-57
Bradi1g58070	Sb02g002650	0.37	0.66	0.57	50.51	4.0E-54
LOC_Os07g04670	GRMZM2G142721	0.30	0.40	0.74	30.93	2.0E-60
LOC_Os01g61310	GRMZM2G359892	0.15	0.51	0.30	39.52	3.0E-79
LOC_Os01g61310	GRMZM2G162109	0.16	0.49	0.33	37.37	7.0E-79
LOC_Os05g39500	GRMZM2G359892	0.20	0.48	0.41	37.29	1.0E-80
LOC_Os05g39500	GRMZM2G162109	0.19	0.41	0.45	31.85	1.0E-80

Table S5: Continued

Gene1	Gene2	Ka	Ks	Ka/Ks	Date(million years)	E-value
Sb09g023120	GRMZM2G135332	0.20	0.57	0.34	43.99	9.0E-79
Sb09g023120	GRMZM2G162109	0.08	0.21	0.38	16.02	1.0E-84
Sb09g016440	GRMZM2G030116	0.08	0.29	0.27	22.25	6.0E-97
Sb03g038670	GRMZM2G162109	0.16	0.33	0.49	25.52	4.0E-70
Sb03g038670	GRMZM2G135332	0.04	0.22	0.17	17.30	2.0E-79
Sb03g038670	GRMZM2G359892	0.17	0.42	0.41	32.47	4.0E-70
Sb02g002650	GRMZM2G142721	0.08	0.15	0.55	11.69	4.0E-92
GRMZM2G095382	GRMZM2G459645	0.03	0.15	0.24	11.34	2.0E-81
GRMZM2G095382	GRMZM2G142721	0.11	0.36	0.30	27.41	4.0E-81
GRMZM2G027302	GRMZM2G168371	0.04	0.25	0.15	18.97	2.0E-94
GRMZM2G034810	GRMZM2G135332	0.06	0.19	0.29	14.48	2.0E-87
GRMZM2G034810	GRMZM2G359892	0.16	0.49	0.32	37.98	1.0E-79
GRMZM2G061499	GRMZM2G095382	0.14	0.46	0.31	35.58	4.0E-77
GRMZM2G061499	GRMZM2G147241	0.03	0.16	0.20	12.50	2.0E-82
GRMZM2G135332	GRMZM2G162109	0.21	0.50	0.42	38.52	2.0E-77
GRMZM2G162109	GRMZM2G359892	0.07	0.22	0.32	17.14	6.0E-84
GRMZM2G135332	GRMZM2G359892	0.19	0.53	0.35	41.09	2.0E-77

Table S6: The conserved motifs identified from ALOG family proteins

Motif No	Motif length	Sequence
Motif 1	61	RYENQKRRDWNTFGQYLRNHRPPLSLSRCSGA HVLEFLRYLDQFGKTKVHTQLCPFFGHPN
Motif 2	26	CPCPLRQAWGSLDALIGRLRAAYEEN
Motif 3	36	PETNPFGARAVRLYLREVRDSQAKARGISYEKKKRK
Motif 4	26	MDLSPNPESPGSGGDGGGGGGGAGGS
Motif 5	19	HHHHHHHHMIPGQEPSAAD
